# Tabak Harm Reduction: Die Notwendigkeit eines Paradigmenwechsels in der deutschen Tabakkontrollpolitik

**DOI:** 10.1007/s00103-024-03900-x

**Published:** 2024-06-04

**Authors:** Larissa Steimle, Meryem Grabski, Heino Stöver

**Affiliations:** Institut für Suchtforschung Frankfurt (ISFF), Fachbereich 4 „Soziale Arbeit und Gesundheit“, Frankfurt University of Applied Sciences, Nibelungenplatz 1, 60318 Frankfurt am Main, Deutschland

**Keywords:** Rauchentwöhnung, E‑Zigaretten, Schadenminderung, Gesundheitspolitik, Rauchen, Smoking cessation, E‑cigarettes, Dual use, Public health policy, Smoking

## Abstract

Der Konsum von Tabak ist nach wie vor das größte vermeidbare Gesundheitsrisiko in Deutschland. Jährlich sterben über 127.000 Menschen vorzeitig an den Folgen des Tabakkonsums – jede fünfte Krebsneuerkrankung ist eine Folge des Rauchens. Während Deutschland im internationalen Vergleich mit der derzeitigen Tabakkontrollpolitik bestehend aus Werbeverboten, einer Förderung von rauchfreien Umgebungen sowie einem alleinigen Abstinenzparadigma nur sehr schleppend eine Veränderung herbeiführt, zeigen uns andere Länder, wie durch eine Integration von „Harm Reduction“ (Konzept der Schadensminimierung) als ergänzende Maßnahme sowie eine deutlich aktivere Unterstützung von ausstiegswilligen Menschen die Zahl der Raucher*innen gesenkt und dadurch Todesfälle verhindert werden können. Dieser Beitrag stellt zunächst die aktuelle Lage sowie die aktuellen Maßnahmen in Deutschland vor. Aus einem Blick in das Vereinigte Königreich, das mit seiner Tabakkontrollpolitik europaweit als Vorreiter gilt, sowie einer Zusammenfassung des aktuellen Forschungsstandes werden Empfehlungen für Veränderungen der derzeitigen Tabakkontrollpolitik in Deutschland abgeleitet.

## Abstract (en)

The consumption of tobacco continues to be the greatest preventable health risk in Germany. Every year, more than 127,000 people die prematurely as a result of using tobacco — one in five new cases of cancer is a consequence of smoking. Germany’s current tobacco control policy, consisting of advertising bans, the promotion of smoke-free environments, and an abstinence-only paradigm, are only able to initiate slow change. Other countries have shown how the number of smokers can be reduced and deaths prevented by integrating harm reduction as a complementary measure and providing a much more active support for people who want to quit. This article first presents the current situation and current measures in Germany. Then recommendations for changes to the current tobacco control policy in Germany are derived from a look at the United Kingdom, which is considered a pioneer throughout Europe with its tobacco control policy, as well as a summary of the current state of research.

## Tabakkontrollpolitik in Deutschland

Deutschland gilt weiterhin als ein Tabakhochkonsumland. Laut der Deutschen Befragung zum Rauchverhalten (DEBRA) beträgt die Prävalenz des Tabakrauchens in der erwachsenen Bevölkerung gegenwärtig 32,4 % [1]. Laut dem Deutschen Krebsforschungszentrum sterben in Deutschland jährlich über 127.000 Menschen vorzeitig an den Folgen des Tabakkonsums. Damit ist der Konsum von Tabak für 13,3 % aller Todesfälle in Deutschland verantwortlich [2] und stellt gemäß dem Bundesministerium für Gesundheit „das größte vermeidbare Gesundheitsrisiko in Deutschland“ dar [3]. Die weitreichenden gesundheitlichen Folgen des Tabakkonsums verursachen enorme gesamtgesellschaftliche Kosten von jährlich 97 Mrd. €. Hinzu kommen weitereichende ökologische Schäden [2].

Vor diesem Hintergrund stellen die Förderung von Motivation zum Rauchausstieg^1^, die Unterstützung von rauchausstiegswilligen Menschen und verhältnispräventive Anstrengungen gesundheitspolitische Herausforderungen in Deutschland dar. Obwohl sich alle weitestgehend darüber einig sind, dass die Rauchprävalenz weiter gesenkt werden sollte, zeigen die aktuellen Prävalenzen, dass Deutschland mit seiner Tabakkontrollpolitik weit hinter anderen Ländern zurückliegt. Besonders deutlich wird dies anhand der „Tobacco Control Scale“, einer Skala von 37 europäischen Ländern zur Quantifizierung der Umsetzung von Maßnahmen zur Eindämmung des Tabakkonsums [4]. Während Länder wie Irland und das Vereinigte Königreich ganz oben auf der Skala liegen, belegt Deutschland derzeit Platz 34 von 37 Plätzen. Vor allem vulnerable Gruppen mit niedrigem sozioökonomischen Status werden mit der aktuellen Tabakkontrollpolitik nicht ausreichend erreicht, was sich an den erhöhten Prävalenzen zeigt [5].

Der Fokus der aktuellen Tabakkontrollpolitik in Deutschland besteht weitestgehend aus einem Verbot von Tabakaußenwerbung, welches sowohl für Verbrennungszigaretten als auch für E‑Zigaretten und Tabakerhitzer gilt, einer Besteuerung ebendieser Produkte (wobei diese von der Weltgesundheitsorganisation (WHO) als zu lasch kritisiert wurde [6]), Maßnahmen zum Nichtraucher*innenschutz, zum Jugendschutz und zur Produktregulierung sowie Aufklärungskampagnen („Be Smart – Don’t Start“ [7]). Darüber hinaus ist eine Übernahme der Kosten bei der Teilnahme an einem evidenzbasierten Programm zur Rauchentwöhnung geplant. Letzteres soll allerdings nur einmalig und nur für „Versicherte, bei denen eine bestehende schwere Tabakabhängigkeit festgestellt wurde“ [8], zur Verfügung stehen. Unter evidenzbasierten Programmen werden in Deutschland Kurzberatungen, Verhaltenstherapien, verschreibungspflichtige Medikamente oder Telefonberatungen verstanden. Neben diesen Maßnahmen ist eine Vielzahl verschiedener Präparate zur Nikotinersatztherapie (NET) auf dem deutschen Markt erhältlich (z. B. Nikotinpflaster, -kaugummis, -lutschtabletten, -nasenspray). Insgesamt liegt der zentrale Fokus in Deutschland auf der Unterstützung des Rauchausstiegs bzw. einem Ausstieg aus der Nikotinabhängigkeit durch die Linderung von Entzugssymptomen [9]. Auch wenn dieser Fokus generell begrüßenswert ist, scheint dieser Weg nicht ausreichend zu sein, um alle Raucher*innen von Verbrennungszigaretten zu erreichen. Daher braucht es Alternativen für diejenigen, die nicht aufhören wollen oder können. Eine Veränderung der Ausrichtung hin zu Alternativen bietet das Konzept „Harm Reduction“ (dt. Schadensminimierung). Im Folgenden wird dieses Konzept als Ergänzung zur bestehenden abstinenzorientierten Tabakkontrollpolitik diskutiert.

## Tabak Harm Reduction

Untersuchungen mit unterschiedlichsten Forschungsdesigns und Versuchsgruppen zeigen, dass Nikotinersatzprodukte durchaus effektiv sind und beim Rauchstopp helfen [10]. Gleichwohl gelten diese nicht als besonders beliebt, insofern als sie kaum im Rahmen von Rauchstoppversuchen von Raucher*innen genutzt werden [5]. Das ist besonders problematisch, da Versuche, ohne entsprechende Unterstützung, wenig Aussicht auf Erfolg haben [5]. Gleichzeitig zeigen Studien, dass immer mehr Raucher*innen von Verbrennungszigaretten eine Entwöhnung mit der E‑Zigarette versuchen [11]. Gemäß Kotz et al. ist die E‑Zigarette die am häufigsten verwendete Methode in Deutschland, um das Rauchen von Verbrennungszigaretten aufzugeben [5]. E‑Zigaretten sind Geräte, die ein Liquid zu einem Aerosol verdampfen, wobei Liquids ohne und mit Nikotin erhältlich sind [12]. Neben ihrem Einsatz zur Entwöhnung vom Tabakkonsum wird der E‑Zigaretten-Konsum auch im Sinne von „Harm Reduction“, diskutiert. Das Konzept entwickelte sich in den 1980er-Jahren vor allem in Bezug auf Angebote für Konsumierende illegaler Substanzen und galt als Gegenentwurf zu „bevormundenden“ und „entmündigenden“ Begriffen, Konzepten und Praktiken der damals ausschließlich abstinenzorientierten Drogenhilfe [13]. Ein weiteres Produkt, das momentan als potenziell schadensreduzierend diskutiert wird, ist der Tabakerhitzer. Hier kommt echter Tabak zum Einsatz, der auf etwa 350 Grad erhitzt wird, was zu weniger Schadstoffen im Rauch führen soll [12].

Der Einsatz von E‑Zigaretten und Tabakerhitzern zur Entwöhnung von Tabakzigaretten, aber auch als Harm Reduction-Maßnahme wird national wie international kontrovers diskutiert. Diese Produkte sind bisher nicht Teil der deutschen Tabakkontrollpolitik, insofern, als dass sie nicht als Maßnahme genutzt werden. Entsprechend werden sie derzeit in der S3-Leitlinie „Rauchen und Tabakabhängigkeit: Screening, Diagnostik und Behandlung“ auch nicht als evidenzbasierte Maßnahme empfohlen [14]. Diese Leitlinie bietet Gesundheitsfachkräften Empfehlungen und Richtlinien für die Behandlung von Tabakabhängigkeit und wird darüber hinaus als Referenz verwendet, um optimale Behandlungsansätze zu wählen. Nachfolgend wird diskutiert, ob und inwieweit diese alternativen Produkte die bestehenden Tabakkontrollmaßnahmen unterstützen können.

### Schädlichkeit von E-Zigaretten und Tabakerhitzern

Grundsätzlich merken die Kritiker*innen von E‑Zigaretten an, dass auch bei diesen Produkten, durch das enthaltene Nikotin [15] und durch die Liquids bzw. Aerosole [16], von gesundheitsschädlichen Effekten auszugehen ist. Insgesamt ist zudem nach derzeitigem Stand unklar, welche gesundheitlichen Langzeitfolgen aus dem dauerhaften Konsum von E‑Zigaretten resultieren [17]. Anders als E‑Zigaretten sind Tabakerhitzer nicht tabakfrei. Der schadensminimierende Effekt bei der Nutzung von Tabakerhitzern ergibt sich aus den niedrigeren Temperaturen, bei denen der Tabak vaporisiert (verdampft), aber nicht verbrannt wird. Studien zeigen, dass gefährliche Chemikalien auch durch das Vaporisieren mit Tabakerhitzern entstehen, wenn zum Teil auch in niedrigerer Konzentration als bei Verbrennungszigaretten [18]. Auch wenn Tabakerhitzer somit ein nützliches Hilfsmittel zur Schadensminimierung darstellen könnten, gibt es bislang noch nicht ausreichend Studien, um dies zu belegen [19]. Die Befürworter*innen wiederum argumentieren, dass E‑Zigaretten und möglicherweise auch Tabakerhitzer zwar gewisse Risiken mit sich bringen, im Vergleich zur Verbrennungszigarette jedoch weitaus weniger schädlich sind, weshalb ein Umstieg von Raucher*innen auf E‑Zigaretten mit einer vielfach geringeren gesundheitlichen Belastung einhergeht [19–22].

### Dual-use

Ein weiterer Punkt der Kritiker*innen ist der Verweis auf den sogenannten Dual-use [23]. Damit ist gemeint, dass neben dem E‑Zigarettenkonsum weiterhin Tabakzigaretten geraucht werden. 2017 wurde Dual-use von 74,5 % der E‑Zigarettennutzer*innen praktiziert [12]. An dieser Stelle sei allerdings darauf hingewiesen, dass zwischen 2 Ansätzen unterschieden werden sollte: Add-on-use (E-Zigaretten werden zusätzlich zu einem weitgehend unveränderten Konsum an Verbrennungszigaretten konsumiert) und Dual-use (Verbrennungszigaretten werden durch die E‑Zigarettennutzung zu einem gewissen Grad ersetzt). Letzteres führt gemäß Storck zu einer reduzierten Schadstoffaufnahme [23]. Während also ein Add-on-use nicht zu empfehlen ist, da der Schaden nicht reduziert wird und mit einem verstärkten Wunsch nach Nikotin einhergeht, kommt es beim Dual-use zu einer Reduktion der Schadstoffe, bei unverändertem Bedarf nach Nikotin [23]. Darüber hinaus ist Dual-use häufig als Übergangsphase vom ausschließlichen Rauchen zum ausschließlichen Dampfen zu sehen [23].

### E-Zigaretten als Einstieg in das Tabakrauchen?

Gerade im Hinblick auf Jugendliche befürchten Kritiker*innen, dass E‑Zigaretten einen Einstieg in das Tabakrauchen darstellen könnten [24]. Bisher zeigt sich, dass Produkte wie E‑Zigaretten, Tabakerhitzer und Wasserpfeifen gerade unter jungen Erwachsenen sehr beliebt sind [25]. Daten aus Deutschland zeigen, dass im Jahr 2021 6,1 % der 12- bis 17-jährigen Befragten und 29,8 % der 18- bis 25-jährigen Befragten Tabakzigaretten rauchten. Grundsätzlich ist in beiden Altersgruppen das Rauchen rückläufig, wobei der Rückgang unter den 18- bis 25-Jährigen ab 2014 stagnierte [26]. Der Konsum von Wasserpfeifen ist in beiden Altersgruppen rückläufig, ebenso wie der Konsum von E‑Zigaretten (seit 2018). In der Altersgruppe der 12- bis 17-jährigen Jugendlichen ist der Konsum von E‑Shishas zurückgegangen, während er bei den 18- bis 25-jährigen steigt. Der Konsum von Tabakerhitzern ist in beiden Altersgruppen leicht angestiegen [26]. Gemäß der DEBRA-Studie haben unter den 14- bis 17-Jährigen, die E‑Zigaretten konsumieren, 38,9 % noch nie Tabak konsumiert. Als Grund für den E‑Zigarettenkonsum nennen 71,2 % „Spaß“, weitere genannte Gründe sind der Geschmack und die „Coolness“ [12].

Insgesamt ist aus Studien ersichtlich, dass E‑Zigaretten nur sehr selten von Personen konsumiert werden, die noch nie Tabak geraucht haben [12]. Daten aus dem Vereinigten Königreich zeigen jedoch, dass der Anteil der Kinder und Jugendlichen (11–17 Jahre), die mit Dampfen experimentieren, im März/April 2023 im Vergleich zum Vorjahr um 50 % gestiegen ist [27]. Gleichzeitig ist der Marktanteil von Einweg-E-Zigaretten im selben Zeitraum angestiegen [27]. Genau wie in Deutschland sind die Prävalenzen von Tabakraucher*innen – zumindest im Vereinigten Königreich [28, 29] – rückläufig. Es lässt sich festhalten, dass regelmäßige E‑Zigaretten-Konsument*innen meist Erwachsene sind, die bereits rauchen, und es derzeit keine Beweise dafür gibt, dass E‑Zigaretten die Zahl der Raucher*innen erhöhen [30].

### E-Zigaretten zur Tabakentwöhnung?

Befürworter*innen des Einsatzes von E‑Zigaretten zur Tabakentwöhnung argumentieren, dass durch die ähnliche Haptik von E‑Zigaretten und Verbrennungszigaretten (vor allem im Vergleich zu NET) und durch die ähnlich schnelle Nikotinaufnahme ins Gehirn ein Rauchausstieg erleichtert werden könnte. Eine repräsentative Umfrage aus Deutschland zeigt, dass die Verwendung von E‑Zigaretten beim Versuch, mit dem Rauchen aufzuhören, mit einer höheren Abstinenzrate verbunden ist, als dies bei Verwendung von NET oder ohne Hilfe der Fall ist [31]. Auch in einer kürzlich veröffentlichten randomisierten klinischen Studie zeigten E‑Zigaretten eine deutlich bessere Wirksamkeit bei der Tabakentwöhnung als herkömmliche NET und eine vergleichbare Wirksamkeit zu dem Medikament Vareniclin [32]. Diese Erkenntnisse decken sich zudem mit einem jüngst erschienenen Cochrane-Review [22]. Kritiker*innen hingegen merken an, dass hierdurch die Gefahr besteht, dass die Entwöhnung schwieriger wird und Raucher*innen von Verbrennungszigaretten, durch die weiterhin bestehende Nikotinabhängigkeit, an ein anderes Produkt gebunden werden. Damit legen Kritiker*innen ein stärkeres Gewicht auf die negativen Folgen von Abhängigkeit an sich. Studien aus den USA und England zeigen, dass der Anstieg des E‑Zigarettenkonsums mit einem statistisch signifikanten Anstieg der Rauchentwöhnungsrate verbunden ist [11, 33].

Zusammenfassend deuten die bisherigen Ergebnisse darauf hin, dass E‑Zigaretten deutlich weniger schädlich sind als herkömmliche Zigaretten [19–22], weshalb die Zahl der Befürworter*innen von E‑Zigaretten entweder zur Unterstützung eines Rauchausstiegs [20] oder auch als Strategie zur Schadenminimierung [9] stetig wächst. Darüber hinaus könnten Tabakerhitzer eine weitere Harm Reduction-Maßnahme sein, wenn auch mit größerem Risiko für die Gesundheit. Ein Land, welches wie kein anderes ergänzend zu verhältnispräventiven Maßnahmen auf Harm Reduction als Strategie setzt, ist das Vereinigte Königreich. Mit dieser Kombination aus verhältnispräventiven Tabakkontrollmaßnahmen und Harm Reduction konnte das Vereinigte Königreich große Erfolge erzielen, weshalb dessen Tabakkontrollpolitik nachfolgend kurz vorgestellt wird.

## Tabakkontrollpolitik im Vereinigten Königreich

Das Vereinigte Königreich zählt zu den Vorreitern im Umgang mit Verbrennungszigaretten, was sich in einem Rückgang der Rauchprävalenz von 19,8 % im Jahr 2011 auf 13 % im Jahr 2021 widerspiegelt, wobei das Ziel ist, die Raucherprävalenz bis 2030 in allen Gruppen auf unter 5 % zu senken [28, 29]. Ein Grund für diesen Erfolg liegt darin, dass das Vereinigte Königreich Tabak Harm Reduction als Strategie nutzt, was sich in der prominenten Rolle von E‑Zigaretten zeigt, die Teil der Rauchentwöhnungsprogramme sind [34]. Darüber hinaus zeigt sich, dass das Vereinigte Königreich auch in anderen Bereichen, wie z. B. dem Preis, dem Konsumverbot im öffentlichen Raum, Werbeverbote, Warnhinweise, aber auch bei der Unterstützung von Rauchstopps besser als Deutschland abschneidet.

Während somit in Deutschland in der Regelversorgung Potenziale ungenutzt bleiben, indem kaum aktive Versuche unternommen werden, die Rauchprävalenzen zu senken, wird im Vereinigten Königreich Kurzberatung zur Tabakentwöhnung etwa dreimal so häufig angeboten und die leitliniengerechte Kombination aus ärztlicher Kurzberatung und Pharmakotherapie zehnmal so häufig [35]. In Deutschland konsultieren laut DEBRA-Studie 73 % der Raucher*innen einmal im Jahr eine*n Ärzt*in, wobei nach Angaben dieser Raucher*innen bei 81 % der Besuche nicht über das Rauchen gesprochen wird [35]. Damit bestehen nur wenig aktive Versuche, direkt auf Raucher*innen zuzugehen. Kastaun und Kotz (2019) sehen die geringe Beratungsleistung von Ärzt*innen zur Tabakentwöhnung vor allem in strukturellen Barrieren begründet, welche die Umsetzung leitliniengerechter Tabakentwöhnung erschweren, darunter ein Mangel an Zeit, das Fehlen einer Ausbildung in der Durchführung der Tabakentwöhnung, fehlende Überweisungsmöglichkeiten zu Entwöhnungskursen und ein Mangel an Kostenerstattung für das ärztliche Angebot sowie für die Inanspruchnahme evidenzbasierter Therapien der Tabakentwöhnung [35].

Im Vereinigten Königreich können landesweit Beratungsdienste in Anspruch genommen werden, um kostenlos evidenzbasierte Unterstützung beim Rauchstoppversuch zu erhalten [36]. Weiterhin gibt es kostenlose Online-Kurzberatungstrainings für Angehörige medizinischer Berufe [35] und es existiert ein „Quality and Outcomes Framework“ das Ärzt*innen finanziell für die Durchführung evidenzbasierter Rauchstoppinterventionen belohnt [37]. Im Vergleich zum Vereinigten Königreich existiert in Deutschland kein Anreizsystem, evidenzbasierte Therapien werden nur teilweise von den Krankenkassen erstattet und weiterhin ist eine systematische Ausbildung zur Tabakentwöhnung im Curriculum des Medizinstudiums nicht vorgesehen [35]. Im Vereinigten Königreich trägt der Mix aus verhaltens- und verhältnispräventiven Maßnahmen zur Reduktion des Anteils der Tabakraucher*innen bei.

## Schlussfolgerungen für Deutschland

Aus den bisherigen Ausführungen werden nachfolgend Schlussfolgerungen für die Tabakkontrollpolitik in Deutschland formuliert.

### Die Potenziale von E-Zigaretten, Tabakerhitzern etc. nutzen

Die bisherige Tabakkontrollpolitik in Deutschland fokussiert weitestgehend eindimensional auf Nikotinabstinenz. Viele Raucher*innen schaffen diesen Schritt allerdings nicht, weshalb Harm Reduction als Zieloption in die aktuelle Tabakkontrollpolitik aufgenommen werden sollte. Nach allem, was wir wissen, bieten vor allem E‑Zigaretten und möglicherweise auch Tabakerhitzer großes Potenzial sowohl bei der Tabakentwöhnung als auch als Maßnahme zur Schadensminimierung, da sie, nach allem, was wir wissen, die gesundheitliche Situation für Nutzer*innen von Tabakzigaretten zumindest nicht verschlimmern. Darüber hinaus sind E‑Zigaretten bereits jetzt die am häufigsten genutzte Einzelmaßnahme zur Unterstützung der Rauchentwöhnung und das ohne offizielle Empfehlung [38].

Britton et al. (2020) argumentieren, dass eine pauschale Ablehnung von E‑Zigaretten dazu führt, dass Raucher*innen, die durch den Umstieg auf ein risikoärmeres Produkt mit dem Rauchen aufgehört hätten, weiterhin rauchen und dadurch vorzeitig sterben. Zweitens können Menschen, die erfolgreich auf das Dampfen umgestiegen sind, wieder zum Rauchen zurückkehren, wenn sie zu der Überzeugung gelangen, dass das Dampfen keinen gesundheitlichen Nutzen hat. Drittens birgt die Behauptung, dass das Dampfen nicht zur Raucherentwöhnung beitragen kann, die Gefahr, dass das Vertrauen der Öffentlichkeit in die Wissenschaft untergraben wird, obwohl eindeutige Belege dafür vorliegen, dass E‑Zigaretten bei einer Rauchentwöhnung unterstützen können [28]. In der Europäischen Union glauben nur 37 % derjenigen, die E‑Zigaretten zu einem Rauchstoppversuch bzw. zur Rauchreduktion genutzt haben, dass Dampfen weniger schädlich sei als der Tabakkonsum [39]. Diese öffentliche Unsicherheit könnte verhindert werden, wenn klar und deutlich über die aktuelle Evidenz – die eine geringere Schädlichkeit von E‑Zigaretten belegt – aufgeklärt werden würde. Gerade Personen, die nicht mit dem Nikotinkonsum aufhören wollen oder können, sollten E‑Zigaretten als schadensminimierende Maßnahme empfohlen werden. Trotz des nachgewiesenen Nutzens von alternativen Produkten für den Rauchausstieg bleiben Risiken, auf die transparent hingewiesen werden muss. Es sollte weiterhin von einem dauerhaften Konsum von E‑Zigaretten und Tabakerhitzern sowie von einem Add-on-use abgeraten werden.

### Eine aktive, zielgruppenspezifische Tabakkontrollpolitik umsetzen

Der Vergleich mit anderen Ländern hinsichtlich des Anteils von Raucher*innen in der Bevölkerung zeigt, dass die aktuellen Maßnahmen der deutschen Tabakkontrollpolitik (Werbeverbot, Förderung von rauchfreien Umgebungen, alleiniges Abstinenzparadigma usw.) unzureichend sind, auch wenn der Ansatz grundlegend begrüßenswert ist. Es braucht weitergehende Maßnahmen zur Unterstützung von ausstiegswilligen Raucher*innen, darunter der kostenfreie Zugang zu evidenzbasierten Therapien, unabhängig vom sozioökonomischen Status und von der Schwere der Abhängigkeit, sowie die Ausbildung aller in der direkten medizinischen Patientenversorgung tätigen Personen, um Tabakentwöhnung optimal anbieten zu können [35].

Kersting et al. (2021) fordern ein strukturiertes Behandlungsprogramm zur Tabakentwöhnung, wobei alle Interventionen „von der einfachen Ansprache bis zur Gruppentherapie finanziell gefördert“ werden sollten. Weiterhin halten sie, um Hausärzt*innen zu entlasten, ein Ausbau von externen Angeboten wie Tabakentwöhnungskurse oder Raucher*innenambulanzen für wünschenswert [40]. Insgesamt braucht es daher ein aktiveres Zugehen auf die Raucher*innen und eine größere Unterstützung mit evidenzbasierten Methoden. Nach wie vor werden Rauchstoppversuche nur selten durch evidenzbasierte Methoden unterstützt und sind somit oftmals erfolglos [38].

Die evidenzbasierten, kostenfreien Angebote und Maßnahmen sollten weiterhin zielgruppenspezifisch ausgestaltet sein mit einem Fokus darauf, besonders vulnerable Gruppen zu erreichen, wie beispielsweise Personen mit niedrigem sozioökonomischen Status, die besonders betroffen sind [5]. In Bezug auf Jugendliche gilt es, E‑Zigaretten möglichst unattraktiv zu gestalten und diese Gruppe im Besonderen auf die Risiken eines Konsums hinzuweisen. Studien deuten darauf hin, dass mögliche negative Auswirkungen von E‑Zigaretten gerade in dieser Gruppe nicht bekannt zu sein scheinen [41] und vor allem über soziale Medien für E‑Zigaretten geworben wird, wodurch eine Regulierung zusätzlich erschwert ist [42]. Insgesamt braucht es Programme und Maßnahmen, die es erlauben, individuelle Strategien im Umgang mit dem Tabakkonsum zu finden.

Zusammenfassend setzt die Tabakkontrollpolitik in Deutschland vor allem auf verhaltenspräventive Maßnahmen (z. B. rauchfreie Umgebungen), eine Unterstützung von ausstiegswilligen Raucher*innen (allerdings in unzureichendem Maße) sowie eine starke Abstinenzorientierung, ohne Zuhilfenahme von schadensminimierenden Strategien und ohne Blick auf die individuellen Bedürfnisse. Der Wissenschaftsjournalist Jazbinsek (2021) stellte fest: „Kein anderes Land in Europa unternimmt so wenig zur Eindämmung des Tabakkonsums wie Deutschland“ [43], was auch der aktuelle WHO-Bericht gezeigt hat [6]. Aufgrund dessen ist es an der Zeit, den Einsatz von E‑Zigaretten und Tabakerhitzern im Sinne von Harm Reduction und als Hilfsmittel zur Tabakentwöhnung ergänzend zu den bereits bestehenden Maßnahmen der Tabakkontrollpolitik in Deutschland voranzubringen.

## Footnotes

1 Mit „Rauchausstieg“ bzw. „Rauchstopp“ ist in diesem Artikel die Abstinenz von Tabak‑/Verbrennungszigaretten gemeint.
